# Assessment of Different Conventional and Biofortified Wheat Genotypes Based on Biology and Damage Pattern of *Rhyzopertha dominica* and *Trogoderma granarium*

**DOI:** 10.3390/insects16010066

**Published:** 2025-01-11

**Authors:** Hafiz Muhammad Bilal Yousuf, Muhammad Yasin, Muhammad Asif Khan, Asim Abbasi, Muhammad Arshad, Muhammad Anjum Aqueel, Inzamam Ul Haq, Waleed A. A. Alsakkaf, Marwa I. Mackled, Nazih Y. Rebouh, Hayssam M. Ali

**Affiliations:** 1 Department of Entomology, Faculty of Agriculture and Environment, The Islamia University of Bahawalpur, Bahawalpur 63100, Pakistan; 2 Chinese Academy of Tropical Agricultural Sciences, Coconut Research Institute, Wenchang 571339, China; 3 Department of Food Science and Technology, Faculty of Agriculture and Environment, The Islamia University of Bahawalpur, Bahawalpur 63100, Pakistan; 4 Department of Entomology, University of Agriculture, Faisalabad 38040, Pakistan; 5 Division of Biology, Silwood Park Campus, Imperial College London, Ascot SL5 7PY, UK; 6 State Key Laboratory of Ecological Pest Control for Fujian and Taiwan Crops, Key Laboratory of Biopesticides and Chemical Biology, MOE, College of Plant Protection, Fujian Agriculture and Forestry University, Fuzhou 350002, China; 7 Department of Botany and Microbiology, College of Science, King Saud University, Riyadh 11451, Saudi Arabia; 8 Department of Stored Product Pests, Plant Protection Institute, Agriculture Research Center (ARC), Sabahia, Alexandria 21531, Egypt; 9 Department of Environmental Management, Institute of Environmental Engineering, RUDN University, 6 Miklukho-Maklaya St., 117198 Moscow, Russia

**Keywords:** lesser grain borer, khapra beetle, wheat, storage pests, zinc-biofortified, grain damage, weight loss, moisture content

## Abstract

The lesser grain borer, *Rhyzopertha dominica* (F.) (Coleoptera: Bostrichidae) and khapra beetle, *Trogoderma granarium* E. (Coleoptera: Dermestidae) are primary stored-grain insect pests. Differences in certain biological and physical parameters of both pest species and wheat genotypes were investigated under laboratory conditions. Zinc (Zn)-biofortified (Zincol-2016 and Akbar-2019) and conventional (Arooj-2022, Nawab-2021, Dilkash-2021, Bhakkar Star-2019) wheat genotypes were used in this study. Zn-biofortified genotypes outperformed the conventional ones, with significant differences observed in fecundity, percent adult emergence, total developmental duration, percent grain damage, and weight loss of both insect species. The results further revealed that the fecundity of *R. dominica* and *T. granarium* were lowest on Akbar-2019 in both the free-choice test (42.50 and 33.17) and no-choice test (35.50 and 32.50), respectively. Similarly, percent adult emergence of both insect species was also lowest on Akbar-2019 in both the free-choice test (69.78 and 70.28%) and no-choice test (67.38 and 70.71%). The total developmental period also showed significant variation among the tested genotypes. The longest developmental period was recorded in Akbar-2019, i.e., 44.33 and 58.83 days, for *R. dominica* and *T. granarium*, respectively. Similarly, percent grain damage (13.23 and 10.33%) and weight loss (3.62 and 2.12%) were found to be minimum in Akbar-2019 for both pest species, respectively. Additionally, a positive correlation was observed between grain moisture content and damage parameters, suggesting that the higher moisture content may aggravate the percent grain damage and weight loss. These findings indicate that the nutritional qualities of Zn-biofortified wheat genotypes negatively affected the development of both insect species; thus, it can be an efficacious approach not only for ensuring food security but also for protecting grains against storage pests.

## Simple Summary

This study assessed the effectiveness of conventional and zinc-biofortified wheat genotypes in resisting two major stored-grain pests, the lesser grain borer (*Rhyzopertha dominica*) and the khapra beetle (*Trogoderma granarium*). Under laboratory conditions, researchers evaluated pest reproduction, development, grain damage, and weight loss across different wheat genotypes. The findings revealed that zinc-biofortified wheat genotypes, such as Akbar-2019, were significantly more resistant to these pests compared to conventional types. The resistant varieties exhibited lower pest reproduction and slower development, resulting in reduced grain damage and weight loss. Furthermore, this study highlighted a strong correlation between higher grain moisture content and increased pest activity, suggesting the need for better storage practices. By promoting zinc-biofortified wheat, this research offers the dual benefit of improving nutritional quality and minimizing post-harvest losses, contributing to food security and sustainable agriculture. This approach also provides an environment-friendly alternative to chemical pesticides for the management of stored-product pests.

## 1. Introduction

Global food security is threatened by different primary and secondary stored-grain insect pests that cause severe losses during grains’ processing and storage [1,2,3]. The main reasons behind infestations are mishandling during storage, higher grain moisture content, poor storage structures, and preferred climatic conditions for insect survival and reproduction [4,5]. These losses account for 9% in developed countries, whereas developing nations experience >20% losses in storage [5,6]. Infestation of these storage pests not only causes quantitative losses by feeding but also lowers the quality of grains by their excretions, exuviae, and dead bodies [7,8].

Among the storage pests, the lesser grain borer, *Rhyzopertha dominica* (F.) (Coleoptera: Bostrichidae), and the khapra beetle, *Trogoderma granarium* E. (Coleoptera: Dermestidae), are known to be very serious pests of stored grains [9,10]. Both pest species are classified as secondary pests that cause direct damage to grains by penetrating the seed coat or feeding on sound kernels [11]. *Rhyzopertha dominica* is a cosmopolitan and serious pest of wheat and other grains worldwide. Adults and larvae cause serious damage by feeding inside the grains and producing feces [12]. It is usually found in flour mills, grain markets, food stores, grain elevators, and grain storage facilities at the domestic level [13]. On the other hand, *T. granarium* is a polyphagous pest, attacking many stored products such as grain and cereals, and is recognized as an A2 quarantine pest by the EPPO and ranked as one of the 100 worst invasive species worldwide [1,14]. The larval stage of *T. granarium* is the most destructive stage, however, adults are harmless and not able to feed [15]; warehouses, breweries, mills, and domestic storage facilities are its common habitats [13].

Management of the aforementioned storage pests still relies on the use of synthetic chemical pesticides and fumigants, often applied prophylactically [16]. Although these application methods are more viable in terms of costs involved and efficacy, they are still neglected due to various health and ecological consequences [17]. Furthermore, continuous exposure to these chemicals also triggers resistance development in storage insect pests [16,18]. Moreover, these insects are difficult to manage with chemical insecticides applied directly to grains as most of their life stages remain hidden within the grain commodities [19].

The compromised efficacies of synthetic chemicals demand sustainable and eco-friendly alternatives for the management of these storage insect pests [20,21]. The potential of resistant varieties has been evaluated to suppress the population of stored-grain pests in post-harvest storage structures [22,23]. All stored-grain pests exhibit the phenomenon of preference or non-preference for grains of different varieties [24]. Resistant genotypes with antibiosis mechanisms may affect the survival rate, weight, reproductive potential, and longevity of insects, or they may have an indirect effect by raising the exposure of the insect to its natural enemies because of prolonged developmental time [20,25,26]. Besides this, resistant genotypes also produce secondary metabolites such as terpenes and phenolics, which repel storage insects by exerting toxic effects on them [27]. Thus, resistant varieties have become a crucial element in the success of many ongoing insect pest management programs, serving as an effective, feasible, economical, and environmentally safe pest management approach [28].

Likewise, the role of micronutrients in defense against insects is predominantly documented for zinc (Zn), magnesium (Mn), copper (Cu), and iron (Fe) [29,30]. As a first line of defense, the nutritional status can determine plants’ susceptibility to pests and pathogens [31]. Concentrations of these elements in plants are enhanced through conventional breeding or biotechnological methods to combat insect pests and diseases and to enhance the nutritional profile of grains [32]. Zn is a major player in both plant and animal immune responses [33]. A genetically modified wheat genotype designed for insect pest management is characterized by the integration of two key features: biofortification and resistance to insects [34]. Thus, in addition to biofortification, Zn-biofortified wheat varieties also possess inherent resistance to insect pests [35].

In Pakistan, there is a dire need to conduct more research on biofortified and other resistant wheat genotypes to evaluate the resistance potential against different insect pests. The identification and characterization of traits associated with insect resistance are very important for the development and identification of resistant genotypes [36]. Therefore, current research efforts were focused on assessing the performance of different wheat genotypes, including Zn-biofortified, to evaluate their resistance against *R. dominica* and *T. granarium* by analyzing their biology and damage parameters. Moreover, the incubation period, larval and pupal duration, total developmental period, and male and female longevity of both insect species were also checked on tested wheat genotypes. Finally, simple linear regression analysis was performed to explore the relationships between grain moisture content and the damage parameters of grains.

## 2. Materials and Methods

The present investigations were carried out during April–August 2024 in the Stored Grain Research Laboratory, Department of Entomology, Faculty of Agriculture and Environment, The Islamia University of Bahawalpur, Pakistan (29.3544° N, 71.6911° E). All experiments were carried out at 29 ± 2 °C, 65 ± 5% relative humidity (r.h.), and a photoperiod of 12:12 (L:D) hours.

### 2.1. Wheat Genotypes

Healthy, uninfested, and pesticide-free grains of six wheat genotypes comprising two Zn-biofortified (Zincol-2016 and Akbar-2019) with Zn content and four conventional wheat genotypes (Bhakkar Star-2019, Dilkash-2021, Nawab-2021, and Arooj-2022) were obtained from the Regional Agriculture Research Institute (RARI), Bahawalpur, Pakistan (29.3918° N, 71.6535° E). The zinc content in both Zn- biofortified wheat genotypes was approximately 35 and 37 mg/kg, respectively, which is 26–30% higher than in conventional genotypes [37,38]. Before commencing this study, all wheat genotypes were placed in an oven at 50 °C for 4 h to disinfect the grains. Moreover, the moisture content of all wheat genotypes was maintained below 13% [39]. Following that, a one-kilogram sample from each variety was taken and prepared by sieving through 3/8-, 3/16-, 1/8-, and 1/12-inch mesh sieves to remove impurities. The moisture content of all the samples was determined by a calibrated moisture meter (Dickey-John Multigrain CAC II, Dickey-John Co., Auburn, IL, USA).

### 2.2. Insect Cultures

Populations of *R. dominica* and *T. granarium* were sourced from different areas of Bahawalpur, including grain markets and flour mills. The adults of both species were separated using a camel hair brush, introduced in separate glass jars (1 L capacity; 10 × 18 cm: D × H), and covered with a muslin cloth, kept in place by rubber bands to prevent insects escaping. The laboratory culture of both species was maintained on whole wheat at 29 ± 2 °C, 65 ± 5% r.h., and a photoperiod of 12:12 (L:D) hours in an incubator (FTE 90E VELP Scientifica, Usmate Velate, Italy).

### 2.3. Sex Identification

Since it was difficult to distinguish males and females of *R. dominica* on a morphological basis, a pair of newly emerged (2–4 days old) adults were taken in vials. The vials in which adults copulated were selected and marked as pairs of opposite sexes. During copulation, the female remains underneath the male, and thus, both sexes could easily be separated in different Petri plates marked with male and female; therefore, the measurement of individuals of both sexes was carried out separately. In the case of *T. granarium*, the sexes were identified based on their morphological features. Males are usually 1.4–2.3 mm long and 0.75–1.1 mm wide, while females are 2.1–3.4 mm long and 1.7–1.9 mm wide [40]. The male is dark brownish-black with distinct reddish-brown markings on the elytra and is usually darker than the female [41].

### 2.4. Orientation Tests

Two test methods, free-choice and no-choice tests, were used in the screening of different wheat genotypes for resistance against *R. dominica* and *T. granarium* under laboratory conditions.

#### 2.4.1. Free-Choice Test

In the free-choice test, all the genotypes were subjected to the attack of *R. dominica* and *T. granarium* freely. Twenty grams of grain of each genotype was kept in 50-milliliter-capacity plastic jars (4 × 4 cm: D × H) and arranged in a circular form in a wide plastic tray (35 × 35 × 10 cm: L × W × H), and the insects were allowed to select the potential host. There were six jars, and each jar represented a single replication for each insect species. Ten adult pairs (24–48 h old) of *R. dominica* and *T. granarium* were taken from the laboratory culture and released in each jar separately. The jars were kept in an incubator at the conditions mentioned above. The insects were allowed to remain there for mating and oviposition for one week and were then removed. The number of eggs laid by the adult females on different genotypes was counted under a 10 × hand lens after isolating them with the help of a camel hair brush, and hatchability percentage and larval survival rate were determined.

#### 2.4.2. No-Choice Test

In the no-choice test, insects were not given a choice to select the suitable host, i.e., the adults of both species were allowed to access only one genotype. For this test, 10 g of grain from each genotype was placed in separate plastic jars of 50 mL capacity (4 × 4 cm: D × H). Two pairs of newly emerged adults (24–48 h old) of *R. dominica* and *T. granarium* were released in each jar separately. The jars were kept in an incubator at the conditions mentioned above. Released insects were allowed to oviposit for seven days. Subsequently, the insects were removed, and then the same procedure was followed as in the free-choice test. The experiment was replicated six times.

The adults of both species emerged from different jars; both in the free- and no-choice tests were noted daily and removed from the respective jars. Counting was carried out until the emergence was completed, and then the percent adult emergence was calculated. For recording the number of male and female adults, newly emerged adults in pairs were placed in transparent plastic specimen tubes and observed on the basis of their copulation. Those who did not mate were replaced by others. This process was repeated up to three times to find out the exact pair of both sexes and then counted to find out the exact sex ratio. The formula suggested by Dobie [42] was used to calculate the susceptibility index of each variety:

$$\mathrm{Susceptibility}\;\mathrm{index}=\frac{\mathrm{Natural}\;\log f\;\times \;100}{D\;}\;\times \;100$$

where f = number of adults that emerged, D = mean developmental period.

The growth index of each genotype was determined by dividing the percentage of adult emergence by the total developmental period [43].

$$\mathrm{Growth}\;\mathrm{index}=\frac{\mathrm{Percent}\;\mathrm{adult}\;\mathrm{emergence}}{\mathrm{Total}\;\mathrm{developmental}\;\mathrm{period}}$$

### 2.5. Percent Grain Damage and Weight Loss

For determination of the percent grain damage, 20 g of grain from each genotype was weighed on an analytical weighing balance (ELB 300, Shimadzu, Kyoto, Japan). The number of grains per 20 g was counted and poured into a 500-milliliter-capacity (8 × 15 cm: D × H) plastic jar, making one replication. There were six such jars to make six replicates for each insect species. Fifty adults (the damaging stage) of *R. dominica* were released into each jar. After 30 days of this experimental set-up, the jars were opened, the damaged grains were isolated, and the number of grains counted. Grains that insect pests had pierced were referred to as damaged grains. The same procedure was adopted for *T. granarium*, in which larvae (2nd instar) were used in place of adults, as used for *R. dominica* [44]. The following formula was used to determine the percentage of grain damage:

$$\mathrm{Percent}\;\mathrm{grain}\;\mathrm{damage}=\frac{\mathrm{Number}\;\mathrm{of}\;\mathrm{damaged}\;\mathrm{grain}s}{\mathrm{Total}\;\mathrm{number}\;\mathrm{of}\;\mathrm{grain}s}\;\times \;100$$

For assessment of weight loss, the number and weight of damaged and undamaged grains were recorded. Afterward, these findings were put into the formula mentioned below:

$$\mathrm{Percent}\;\mathrm{weight}\;\mathrm{loss}=\frac{\left(\mathrm{Nd}\;\times \;\mathrm{Wu}\right)-\left(\mathrm{Wd}\;\times \;\mathrm{Nu}\right)}{(\mathrm{Nd}+\mathrm{Nu})\;\times \;\mathrm{Wu}}\;\times \;100$$

where Nd = total number of damaged grains, Nu = total number of undamaged grains, Wu = weight of undamaged grains, Wd = weight of damaged grains.

Following data collection, jars were again placed at respective places in the experimental set-up. Healthy grains were returned to their jars for subsequent data recordings after 60 and 90 days.

### 2.6. Frass Weight (g)

While determining weight loss, the weight of exuviae, grain dust, dead as well as alive adults, the immature stages of both insect species, and those of other excretions produced during infestation were measured and collectively termed as the frass weight of the respective sample of each genotype.

### 2.7. Determination of Moisture Content (%)

Grain moisture content was determined to investigate its correlation with the levels of insect infestation, healthy and damaged grains, and weight loss. For this purpose, the moisture content of grains was measured before and after infestation. A moisture content of less than 13% was verified for all the testing genotypes before starting the experiment [45].

### 2.8. Developmental Period and Adult Longevity

For observing the total developmental period and adult longevity of *R. dominica* and *T. granarium* on different wheat genotypes, 20 g of grains of each accession was filled in 500-milliliter-capacity (8 × 15 cm: D × H) plastic jars. Five pairs (48–72 h old) of *R. dominica* and *T. granarium* were released in each plastic jar and kept under controlled conditions at 29 ± 2 °C, 65 ± 5% r.h., and a photoperiod of 12:12 (L:D) hrs. Released insects were allowed to oviposit for seven days, and were then discarded. To observe the incubation period, ten eggs from each genotype were placed in a Petri dish, and the incubation period was recorded. Later on, the duration of the immature stages (larval and pupal) of both insect species was recorded. The total number of days required to complete their life cycle from incubation to adult emergence on various wheat genotypes was used to calculate the total developmental time. Subsequently, the adult longevity of both males and females of both species was checked and recorded daily for each wheat genotype until the adults died.

### 2.9. Statistical Analysis

The collected data were statistically analyzed by one-way analysis of variance (ANOVA) under complete randomized design (CRD) using GenStat 15th Edition. Prior to analysis, data were tested for normality and conformed, in accordance with the requirements of ANOVA. Since the data were normally distributed, no data transformation was conducted. Means were compared and separated using the Tukey–Kramer HSD test at a 1% significant level. Simple linear regression analysis explored relationships between the moisture content and different damage parameters of grain. Linear regression slopes were tested for significant differences from zero by Sigma Plot version 10.0 [46].

## 3. Results

### 3.1. Free-Choice and No-Choice Tests for R. dominica

The results showed the significant effects of different wheat genotypes on the developmental stages of *R. dominica* both in free- and no-choice tests (Table 1). The highest fecundity was recorded for Arooj-2022 (54.17 eggs), followed by Dilkash-2021 (51.33 eggs) in the free-choice test, whereas in the no-choice test, it was highest in Dilkash-2021 (46.83 eggs), followed by Arooj-2022 (44.00 eggs). In contrast, the lowest fecundity was noted in Akbar-2019, i.e., 42.50 and 35.50 eggs in the free- and no-choice tests, respectively. Maximum hatching was recorded in Dilkash-2021 (78.24%) during the free-choice test, whereas in the no-choice test, the highest hatching was recorded in Arooj-2022 (76.88%). Contrarily, minimum hatching was recorded in Akbar-2019, i.e., 66.63 and 64.31% in both the free- and no-choice tests, respectively (Table 1).

Larval survival and adult emergence were also more pronounced in Arooj-2022 during both orientation tests. In the free-choice test, larval survival and adult emergence were 81.76 and 82.58%, respectively, while in the no-choice test, it was recorded at 79.39 and 80.72%, respectively. Minimum larval survival and adult emergence were recorded in Akbar-2019, i.e., 69.98 and 69.78% in the free-choice test, whereas these were 67.25 and 67.38% in the no-choice test. With respect to the adult emergence (male and female), the highest number of adults was counted in Arooj-2022, i.e., 27.67 (males: 12.17 and females: 15.50) and 21.67 (males: 8.17 and females: 13.50), while the lowest number of adults was counted in Akbar-2019, i.e., 13.83 (males: 5.50 and females: 8.33) and 10.33 (males: 4.00 and females: 6.33), for the free- and no-choice tests, respectively (Table 1).

The highest susceptibility index was observed for Arooj-2022 (10.79 and 10.00), while the lowest was for Akbar-2019 (6.52 and 5.79) for both tests, respectively. Regarding the growth index, the insects reared on Arooj-2022 had the highest growth index (2.69 and 2.63), while the lowest was observed on Akbar-2019 (1.73 and 1.67) during free- and no-choice tests, respectively (Table 1).

### 3.2. Free-Choice and No-Choice Tests for T. granarium

Similarly, results demonstrated the significant impacts of different wheat genotypes on the developmental stages of *T. granarium* in both orientation tests (Table 2). The highest fecundity was recorded in Nawab-2021 (46.67 eggs) during the free-choice test, while in the no-choice test, it was recorded in Arooj-2022 (40.83 eggs). In contrast, the lowest fecundity was recorded in Akbar-2019 (33.17 and 32.50 eggs) for both tests, respectively. Maximum hatching was recorded in Dilkash-2021 (77.97%) in the free-choice test and (78.46%) in the no-choice test, while the minimum was recorded in Akbar-2019 (68.30% and 70.20%) for both tests, respectively. Larval survival was maximum in Arooj-2022 (77.74 and 79.92%), while the minimum was recorded in Akbar-2019 (66.84 and 67.30%) in both tests, respectively. Likewise, adult emergence was maximum in Dilkash-2021 (82.16%) during the free-choice test, whereas in the no-choice test, Arooj-2022 exhibited maximum adult emergence (81.40%). On the other hand, minimum adult emergence was observed in Akbar-2019, i.e., 70.28% and 70.71%, for both orientation tests, respectively (Table 2).

In respect to the numbers of adult emergence (male and female), the highest number of adults was counted in Arooj-2022, i.e., 21.17 (males: 10.17 and females: 11.00) and 19.83 (males: 9.33 and females: 10.50), while the lowest number of adults was counted in Akbar-2019, i.e., 10.67 (males: 5.00 and females: 5.67) and 10.84 (males: 5.17 and females: 5.67), for *R. dominica* and *T. granarium* both in free-choice and no-choice tests, respectively. The highest susceptibility index was found in Arooj-2022 (6.55 and 6.42), while the lowest was in Akbar-2019 (4.02 and 4.05) for both tests. Similarly, the insects reared on Arooj-2022 had the highest growth index (1.72 and 1.75), while the lowest was observed on Akbar-2019 (1.20 and 1.20) (Table 2).

### 3.3. Effect of R. dominica on Physical Parameters of the Grains

Arooj-2022 exhibited the highest susceptibility towards *R. dominica*, whereas Akbar-2019 displayed the highest resistance during the 90-day storage period (Table 3). The maximum number of grains damaged by *R. dominica* was recorded in Arooj-2022, i.e., 7.33, 37.67, and 146.00 grains, after 30, 60, and 90 days of storage, respectively. Contrarily, the minimum number of damaged grains was recorded in Akbar-2019 after 30 days (1.50 grains), 60 days (9.00 grains), and 90 days (79.67 grains). The highest grain damage percentage was recorded in Arooj-2022 (1.35, 6.91, and 26.84%), whereas it was minimum in Akbar-2019 (0.25, 1.49, and 13.23%) after 30, 60, and 90 days, respectively. Frass weight was also the maximum in Arooj-2022 (0.09, 0.18, and 0.80 g), while the minimum was found in Akbar-2019 (0.03, 0.07, and 0.50 g) after 30, 60, and 90 days, respectively. In the case of weight loss, Arooj-2022 exhibited the highest weight loss (0.66, 2.49, and 7.95%), while the lowest weight loss was recorded in Akbar-2019 (0.10, 0.48, and 3.62%) after 30, 60, and 90 days, respectively (Table 3).

### 3.4. Effect of T. granarium on Physical Parameters of the Grains

The maximum number of damaged grains was recorded in Arooj-2022, i.e., 52.17, 53.67, and 98.17 grains after 30, 60, and 90 days of storage, respectively (Table 4). On the other hand, the minimum number of damaged grains was recorded in Akbar-2019 after 30 days (26.00 grains), 60 days (26.50 grains), and 90 days (61.50 grains). The highest grain damage percentage was recorded in Arooj-2022 (9.41, 9.68, and 17.70%) during all the storage periods, whereas it was minimum in Akbar-2019 (4.37, 4.45, and 10.33%) at 30, 60, and 90 days of exposure, respectively. Frass weight was also maximum in Arooj-2022 (0.22, 0.24, and 0.59 g), while the minimum was observed in Akbar-2019 (0.06, 0.08, and 0.40 g) after 30, 60, and 90 days, respectively. Likewise, the highest weight loss was observed in Arooj-2022 (2.09, 2.10, and 3.93%). Contrarily, Akbar-2019 showed the lowest weight loss (1.17, 1.18, and 2.12%) after 30, 60, and 90 days, respectively (Table 4).

### 3.5. Moisture Content

**Figure 1:**
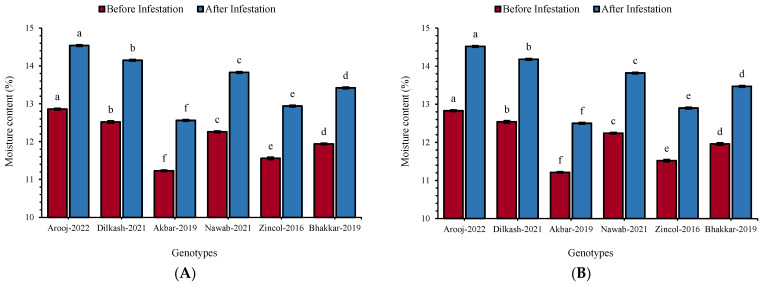
Mean (±SE) moisture content of different wheat genotypes before and after infestation of (**A**) *R. dominica* and (**B**) *T. granarium*. Different letters above the bars show significant differences among the genotypes (Tukey’s HSD test, *p* < 0.01).

The ANOVA revealed significant variations (*p* ≤ 0.01) in grain moisture content among different wheat genotypes before and after infestation. This suggests that the grain moisture content had a significant impact on insect survival, growth, and infestation. Before infestation, Arooj-2022 had the highest moisture content of 12.86 and 12.83% for *R. dominica* and *T. granarium*, respectively, followed by Dilkash-2021 (*R. dominica*: 12.52% and *T. granarium*: 12.54%). Contrarily, the lowest initial moisture content was recorded in Akbar-2019 (*R. dominica*: 11.23% and *T. granarium*: 11.21%), followed by Zincol-2016 (*R. dominica*: 11.55% and *T. granarium*: 11.52%). Significant increases in moisture content of all the wheat varieties were observed after subjecting them to artificial infestation by *R. dominica* (F_5, 35_ = 1751; *p* ≤ 0.01) and *T. granarium* (F_5, 35_ = 1897; *p* ≤ 0.01) for 3 months (May–July). The maximum increase in moisture content was observed in Arooj-2022 (*R. dominica*: 13.11% and *T. granarium*: 13.22%). However, the minimum increase in moisture content was noted in Akbar-2019 (*R. dominica*: 11.83% and *T. granarium*: 11.52%) and Zincol-2016 (*R. dominica*: 11.98% and *T. granarium*: 12.00%) (Figure 1A,B). The increase in moisture content over time was due to the absorbance of atmospheric moisture by the grain, biotic respiration, and insect excretion. The genotypes with the highest initial moisture content exhibited maximum percent infestation and vice versa.

### 3.6. Total Developmental Duration

**Figure 2:**
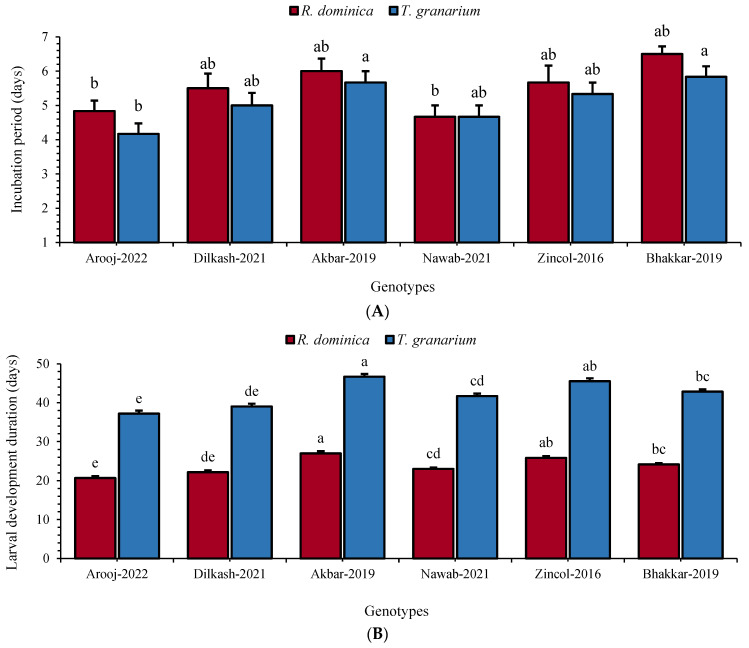
Mean (±SE) incubation period (**A**) and larval development (**B**) of *R. dominica* and *T. granarium* on six wheat genotypes. Different letters above the bars show significant differences among the genotypes (Tukey’s HSD test, *p* < 0.01).

**Figure 3:**
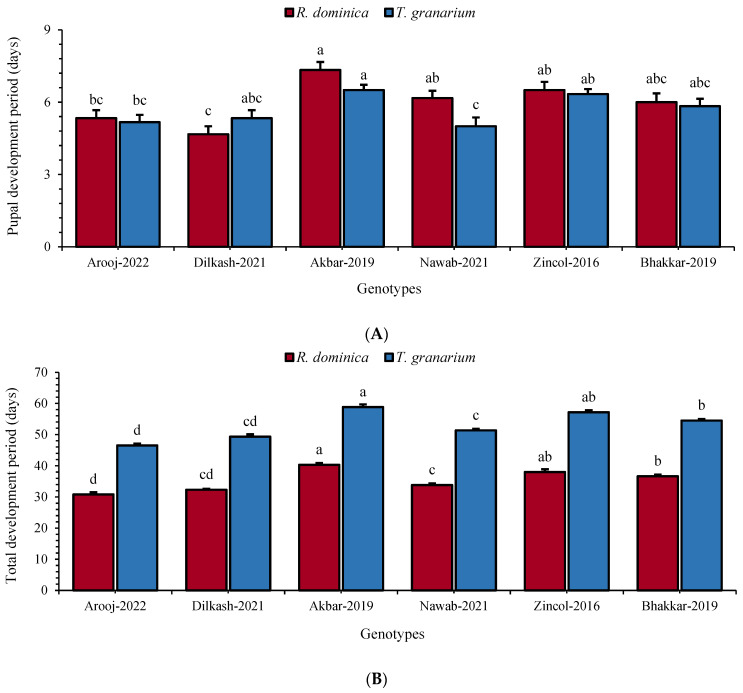
Mean (±SE) pupal (**A**) and total development duration (**B**) of *R. dominica* and *T. granarium* on six wheat genotypes. Different letters above the bars show significant differences among the genotypes (Tukey’s HSD test, *p* < 0.01).

**Figure 4:**
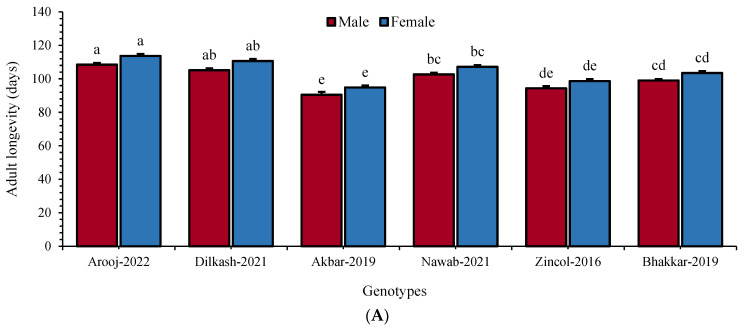

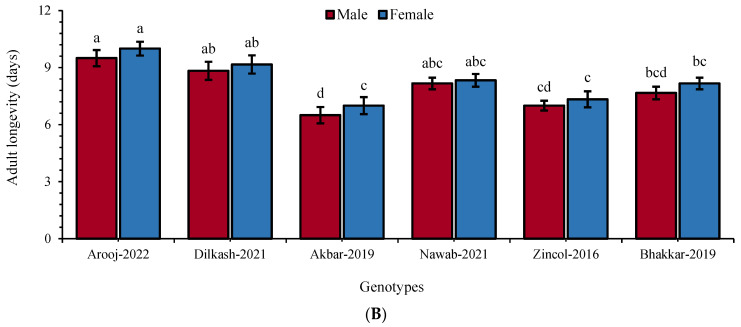
Mean (±SE) male and female longevity of *R. dominica* (**A**) and *T. granarium* (**B**) on six wheat genotypes. Different letters above the bars show significant differences among the genotypes (Tukey’s HSD test, *p* < 0.01).

Based on the significant effects of different genotypes on the biology of *R. dominica* and *T. granarium*, different life history parameters were observed, including egg incubation period, larval, pupal, and total developmental periods, and adult (male and female) longevity (Figure 2, Figure 3 and Figure 4). The observations were made under controlled conditions for both insect species, and it was noted that the egg incubation period was highest in Bhakkar-2019 (*R. dominica*: 6.50 days and *T. granarium*: 5.83 days), followed by Akbar-2019 (*R. dominica*: 6.00 days and *T. granarium*: 5.67 days), respectively. The lowest incubation period was observed in Nawab-2021 (4.67 days) and Arooj-2022 (4.17 days), both for *R. dominica* (F_5, 35_ = 3.54; *p* ≤ 0.01) and *T. granarium* (F_5, 35_ = 3.63; *p* ≤ 0.01), respectively (Figure 2A). The maximum larval period was recorded in Akbar-2019 (*R. dominica*: 27.00 days and *T. granarium*: 46.66 days), followed by Zincol-2016 (*R. dominica*: 25.83 days and *T. granarium*: 45.5 days). In contrast, the minimum larval developmental period was recorded in Arooj-2022 (*R. dominica*: 20.67 days and *T. granarium*: 37.16 days), followed by Dilkash-2021, i.e., *R. dominica*: 22.17 days (F_5, 35_ = 26.3; *p* ≤ 0.01) and *T. granarium*: 39.00 days (F_5, 35_ = 26.6; *p* ≤ 0.01) (Figure 2B).

In the case of pupa, the longest developmental duration was noted in Akbar-2019 (*R. dominica*: 7.33 days and *T. granarium*: 6.5 days). The shortest duration was recorded as 4.67 days in Dilkash-2021 and 5.00 days in Nawab-2021 for *R. dominica* (F_5, 35_ = 7.57; *p* ≤ 0.01) and *T. granarium* (F_5, 35_ = 4.47; *p* ≤ 0.01), respectively (Figure 3A). Total developmental duration from egg to adult was also maximum in Akbar-2019 (*R. dominica*: 40.33 days and *T. granarium*: 58.83 days), followed by Zincol-2016 (*R. dominica*: 38.00 days and *T. granarium*: 57.16 days), while the minimum was recorded in Arooj-2022, i.e., *R. dominica*: 30.83 days (F_5, 35_ = 32.0; *p* ≤ 0.01) and *T. granarium:* 46.5 days (F_5, 35_ = 50.9; *p* ≤ 0.01) (Figure 3B).

### 3.7. Adult Longevity

With respect to adult longevity, a significant difference was recorded in male and female longevity. Overall, females lived for a slightly longer period compared to males. The longest duration (108.5 days) of *R. dominica* males was recorded in Arooj-2022, while that of females was 113.67 days (Figure 4A).

The shortest adult longevity was recorded to be 90.50 and 94.83 days, respectively, both for males (F_5, 35_ = 30.8; *p* ≤ 0.01) and females (F_5, 35_ = 36.9; *p* ≤ 0.01) in Akbar-2019 (Figure 4A). In case of *T. granarium*, the same trend in adult longevity was observed; the longest adult period was observed in Arooj-2022 (male: 9.50 and female: 10.00 days), whereas the shortest male (F_5, 35_ = 8.74; *p* ≤ 0.01) and female (F_5, 35_ = 7.98; *p* ≤ 0.01) longevity was noted in Akbar-2019, i.e., 6.5 and 7.00 days, respectively (Figure 4B).

### 3.8. Correlation Between Grain Moisture Content and Insect Damage Parameters

**Figure 5:**
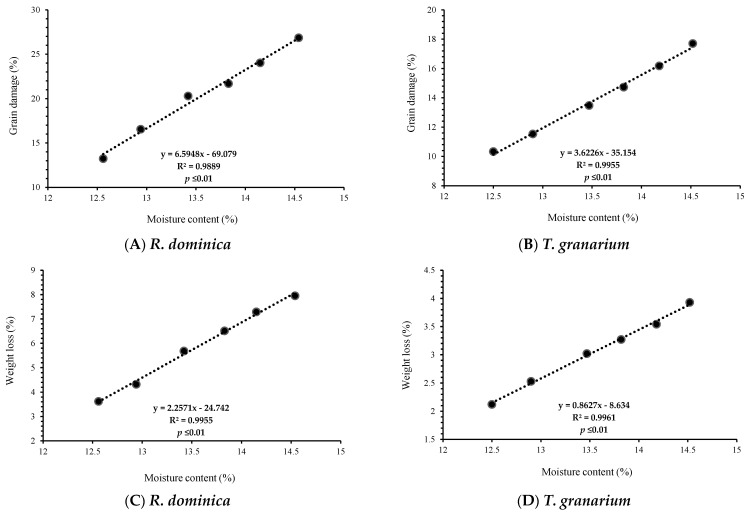
Correlation between grain moisture content and damage parameters caused by *R. dominica* and *T. granarium* on six wheat genotypes. Percent grain damage (**A,B**) and percent weight loss (**C**,**D**).

A simple correlation between grain moisture content and the damage parameters revealed a positive and highly significant correlation (R^2^ ≥ 0.9889 and 0.9955, respectively) between moisture content and percent grain damage for *R. dominica* and *T. granarium* (Figure 5A,B). Similarly, percent weight loss also showed a positive and highly significant correlation (R^2^ ≥ 0.9955 and 0.9961, respectively) with the grain moisture content (Figure 5C,D). The minimum percent grain damage was observed in Akbar-2019 (*R. dominica:* 13.23% and *T. granarium:* 10.33%) with a moisture content of 12.56 and 12.50%, respectively, while the maximum percent grain damage was recorded in Arooj-2022 (*R. dominica*: 26.84% and *T. granarium*: 17.70%) with a moisture content of 14.54 and 14.52%, respectively.

Likewise, minimum percent weight loss was observed in Akbar-2019 (3.62 and 2.12%) with a moisture content of 12.56 and 12.50%, respectively, while the maximum percent weight loss was recorded in Arooj-2022 (7.95 and 3.93%) with a moisture content of 14.54 and 14.52%, respectively.

## 4. Discussion

In the present study, the impacts of Zn-biofortified and conventional wheat genotypes were evaluated in relation to different life history (fecundity, survival, adult emergence, and development duration) and damage parameters (infestation and weight loss) of *R. dominica* and *T. granarium*. The findings of the current study revealed that fecundity, survival, and adult emergence were minimal in the Zn-biofortified genotype Akbar-2019 compared to other tested wheat genotypes. The differences in fecundity, survival rate, and adult emergence observed herein may be attributed to the varied nutritional composition or quality of various secondary compounds in tested wheat genotypes. Moreover, food quality and quantity have a direct effect on the biological parameters of insects [47]. Previously, biological parameters of the red flour beetle, *Tribolium castaneum* (Herbst) (Coleoptera: Tenebrionidae), were assessed on Zn-biofortified and conventional wheat genotypes and reported minimum egg hatching, larval survival, and adult emergence on biofortified wheat [23]. Likewise, *T. granarium* laid a varied number of eggs on various wheat genotypes, and their fertility rates were different [48]. Ebadollahi and Borzoui [25] also evaluated the six rice cultivars and observed a low fecundity and survival rate of *R. dominica* on a rice genotype (Govhar) compared to other rice genotypes. Although the primary objective of biofortification is to address nutritional deficiencies in human diets, at the same time it can indirectly contribute to pest resistance [49,50]. In addition, essential elements like Zinc and Copper play a critical role in creating an antibiosis effect by disrupting pest growth, reproduction, and the survival rate of insects through their impact on the nutritional composition [51,52]. These mechanisms not only enhance the grain resistance to pests but also reduce post-harvest losses and maintain grain quality during storage [23].

Furthermore, the larval, pupal, and total development durations of both insect species were also extended on Zn-biofortified wheat genotypes but remained shorter on other wheat genotypes. In contrast, the male and female longevity of *R. dominica* and *T. granarium* was recorded as shortest in Zn-biofortified wheat, while longest in other conventional wheat genotypes. Previous studies have reported that different mineral nutrients are required in adequate quantities for a healthy lifespan among different insect species [53,54]. As discussed previously, the nutritional quality of the host, secondary metabolites, and quality of food may affect the biology of insects [47]; this might be attributed to the fact that Zn is one of the essential elements required for many biological processes in plants and as a part of defensive mechanisms, which can make biofortified genotypes less favorable for insect feeding and reproduction [55]. Moreover, the addition or alteration of minor nutrients (e.g., Zn) in food changes its nutritional suitability for insects, and may influence their life span [56].

Our findings are also in line with Yasin et al. [3], who reported the lowest development and reproductive rates of *T. castaneum* on Zn-biofortified wheat flour. Syed et al. [57] also studied the life history parameters of *R. dominica* in response to feeding on 25 wheat genotypes and found an extended developmental duration on genotypes GW-366 (35.06 days) and GW-173 (39.80 days). High Zn concentrations negatively affect the growth and development of insects [58]. Our results were also supported by the findings of Golizadeh and Abedi [59], who recorded the lowest fecundity, hatching percentage, and adult longevity of *T. granarium* on the genotypes Kouhdasht and Shirodi as compared to grains of other conventional genotypes. Although these two genotypes along with the rice genotype (Govhar) mentioned above are not Zn-biofortified, they exhibited resistance to insect pests, which can be attributed to their intrinsic traits, including kernel hardness, size, shape, test weight, and the presence of anti-nutritional factors. These characteristics are influenced by the uptake of certain trace elements such as zinc, silicon, copper, and iron [35]. The slow growth and longer developmental time of both insect species in our study suggest that Zn-biofortified wheat genotypes are poor substrates for storage pests.

Storage pests that create holes or tunnels like *R. dominica* and *T. granarium* create more loss than pests that generally feed superficially on grains [44]. With respect to the damage parameters of *R. dominica* and *T. granarium*, our findings showed that percent grain damage and weight loss were higher in conventional wheat genotypes compared to Zn-biofortified wheat genotypes. This effect may be due to the antibiosis properties of Zn, wherein the nutritional composition and biochemical attributes of the grain negatively impact pest physiology. This leads to shorter lifespans and decreased feeding efficiency in pests [60], which directly contributes to less grain damage and reduced weight loss [61,62]. Previous studies suggest that increased nutrient levels in biofortified crops could affect insect behavior positively or negatively, depending on various factors such as the nutritional requirements of the insects and their adaptation to different nutrient levels [35,63].

The current findings are consistent with Wazid et al. [64], who found less seed damage and weight loss by *Callosobruchus analis* (F.) (Coleoptera: Chrysomelidae) and *Sitophilus oryzae* L. (Coleoptera: Dryophthoridae) on Zn-nanoparticles treated sorghum and chickpea seeds, whereas the highest seed damage and weight loss were noticed in the untreated control sample. The results of the present study are also in accordance with the findings of Gami et al. [65], who observed the least grain damage and weight loss in genotype N-813, followed by N-809, compared to the other genotypes. Similarly, the weight of frass produced as a result of damage to grains and the percent weight loss of grains can be ranked with regard to the genotypes as Arooj-2022 > Dilkash-2021 > Nawab-2021 > Bhakkar-2019 > Zincol-2016 > Akbar-2019. In our case, frass production was higher in the wheat genotypes with high grain damage. The same findings were reported by Hassan et al. [44], who stated that the frass weight is directly proportional to the percent grain damage and weight loss.

Moisture content in stored grain is a critical factor that affects its quality, safety, and market value [66]. When moisture levels in stored grains are high, it attracts more pests, leading to increased grain damage and weight loss [67,68]. Insects such as *R. dominica* and *T. granarium* prefer moist environments for reproduction and survival. In our case, moisture content differed significantly among the tested genotypes before and after infestation. All the tested genotypes showed a significant increase in moisture content after subjecting them to artificial infestation with *R. dominica* and *T. granarium* for a storage period of 90 days. These results confirmed the findings of Ahmedani et al. [69], who reported a minimum increase in the moisture content of different wheat genotypes after khapra beetle infestation. Jood et al. [70] observed that the 75% infestation level of *T. granarium* and *R. dominica* caused a significant increase in the moisture content of the infested grains, as seen in the present investigations. The increase in moisture content over time may be due to the absorbance of atmospheric moisture by the grain, biotic respiration, as well as insect excretion [71].

In our case, a positive correlation of grain moisture content with grain damage and weight loss was observed; this might be attributed to the fact that higher moisture content in wheat genotypes creates favorable conditions for storage pests and correlates positively with grain damage and weight loss [69]. Current findings are in accordance with Chatha et al. [72], who noted the highest weight loss in rice genotypes due to high moisture content. Similarly, Wang et al. [73] also reported high weight loss in wheat grain due to high moisture content and temperature.

Overall, a precise knowledge of biofortified wheat genotypes and their interaction with stored-grain insect pests is important to explore to determine what pests could play an important role in expanding storage pest IPM programs. The comparison of some life history traits on conventional and Zn-biofortified wheat genotypes revealed that the insects, *R. dominica* and *T. granarium*, reared on Zn-biofortified genotypes had prolonged larval, pupal, and total development durations. Finally, these genotypes negatively affect fertility by reducing egg production and hatching rates and proved relatively unsuitable hosts for the feeding and development of *R. dominica* and *T. granarium*. Our results provide a theoretical basis for future exploration of the effect of Zn on storage insect growth and development. To investigate the fundamental processes explaining the observed effects, more research is recommended for assessing the impacts of Zn-biofortified wheat genotypes in integrated pest management programs, with a focus on long-term sustainability and pest resistance.

## 5. Conclusions

In this study, conventional and Zn-biofortified wheat genotypes were assessed against *R. dominica* and *T. granarium*. Zn-biofortified wheat showed higher resistance to both insect species than conventional ones, indicating their potential to improve grain preservation during storage. Our findings emphasize the potential of biofortified wheat in not only addressing nutrient deficiencies but also playing an important role in reducing post-harvest losses caused by stored-grain insect pests. These results indicate that Zn-biofortified wheat can provide dual advantages, contributing to both food security and maintaining grain quality in areas where storage losses can severely affect food supply. Further investigation of the genetic and biochemical traits associated with resistance may prove valuable in future wheat breeding programs, potentially leading to the development of cultivars that combine high nutritional value with increased pest resistance, thereby managing the increasing pest pressure. Additionally, it will promote sustainable agriculture and the maintenance of grain storage facilities.

## Figures and Tables

**Table 1:** Mean (±SE) fecundity, hatching rate, larval survival, adult emergence, no. of adults (males and females), susceptibility index, and growth index of *Rhyzopertha dominica* in six wheat genotypes during the free- and no-choice tests. Means in a column followed by different letters show significant differences (*p* < 0.01; Tukey’s HSD test) between the genotypes.

Test Type	Genotypes	Fecundity	Hatching (%)	Larval Survival (%)	Adult Emergence (%)	No. of Males	No. of Females	Susceptibility Index	Growth Index
Free-choice test	Arooj-2022	54.17 ± 0.60 a	75.67 ± 0.96 ab	81.76 ± 1.17 a	82.58 ± 0.46 a	12.17 ± 0.31 a	15.50 ± 0.34 a	10.79 ± 0.24 a	2.69 ± 0.07 a
	Dilkash-2021	51.33 ± 0.49 ab	78.24 ± 0.60 a	79.28 ± 0.88 ab	79.58 ± 0.70 ab	10.83 ± 0.40 a	14.50 ± 0.22 a	10.00 ± 0.07 b	2.46 ± 0.02 b
	Akbar-2019	42.50 ± 0.76 e	66.63 ± 0.84 e	69.98 ± 0.53 e	69.78 ± 0.71 e	5.50 ± 0.34 c	8.33 ± 0.21 d	6.52 ± 0.12 f	1.73 ± 0.04 d
	Nawab-2021	49.00 ± 0.86 bc	73.50 ± 0.75 bc	76.86 ± 1.06 bc	77.12 ± 0.51 bc	8.67 ± 0.33 b	12.67 ± 0.21 b	9.05 ± 0.13 c	2.28 ± 0.04 b
	Zincol-2016	44.83 ± 0.60 de	69.11 ± 0.86 de	72.07 ± 0.65 de	72.35 ± 0.80 de	6.50 ± 0.22 c	9.50 ± 0.34 d	7.34 ± 0.14 e	1.91 ± 0.03 cd
	Bhakkar-2019	47.67 ± 0.76 cd	71.01 ± 0.67 cd	74.37 ± 0.99 cd	74.90 ± 0.93 cd	8.0 0± 0.26 b	10.83 ± 0.31 c	8.01 ± 0.13 d	2.05 ± 0.03 c
	F*_Statistic_*	37.8	29.7	23.9	44.8	64.2	103	121	68.4
	*p_value_*	≤0.01	≤0.01	≤0.01	≤0.01	≤0.01	≤0.01	≤0.01	≤0.01
	HSD at 0.01	2.97	3.39	3.91	3.02	1.36	1.20	0.64	0.19
	%CVs	3.51	2.66	2.94	2.26	9.00	5.75	4.20	4.85
No-choice test	Arooj-2022	44.00 ± 0.63 ab	76.88 ± 1.09 a	79.39 ± 1.18 a	80.72 ± 1.78 a	8.17 ± 0.31 a	13.50 ± 0.43 a	10.00 ± 0.32 a	2.63 ± 0.11 a
	Dilkash-2021	46.83 ± 0.48 a	73.73 ± 1.48 ab	76.33 ± 2.03 ab	77.90 ± 1.79 ab	8.00 ± 0.37 a	12.50 ± 0.43 a	9.34 ± 0.13 a	2.41 ± 0.07 ab
	Akbar-2019	35.50 ± 0.76 e	64.31 ± 0.70 e	67.25 ± 1.26 d	67.38 ± 1.56 c	4.00 ± 0.26 d	6.33 ± 0.33 d	5.79 ± 0.08 d	1.67 ± 0.03 e
	Nawab-2021	41.17 ± 0.79 bc	71.25 ± 0.52 bc	74.43 ± 0.51 abc	75.67 ± 2.01 ab	6.50 ± 0.22 b	10.00 ± 0.37 b	8.29 ± 0.18 b	2.24 ± 0.08 bc
	Zincol-2016	37.83 ± 0.60 de	66.96 ± 0.76 de	69.67 ± 1.05 cd	70.79 ± 1.41 bc	5.00 ± 0.26 cd	7.50 ± 0.34 cd	6.66 ± 0.16 c	1.87 ± 0.06 de
	Bhakkar-2019	39.67 ± 0.76 cd	69.32 ± 0.61 cd	72.21 ± 1.04 bcd	73.05 ± 1.27 bc	5.50 ± 0.22 bc	9.00 ± 0.26 bc	7.30 ± 0.13 c	1.99 ± 0.04 cd
	F*_Statistic_*	36.7	24.6	12.4	8.59	36.3	58.9	77.9	25.4
	*p_value_*	≤0.01	≤0.01	≤0.01	≤0.01	≤0.01	≤0.01	≤0.01	≤0.01
	HSD at 0.01	2.93	3.95	5.42	7.12	1.19	1.57	0.79	0.31
	%CVs	4.08	3.20	4.22	5.46	10.96	9.09	5.67	8.14

**Table 2:** Mean (±SE) fecundity, hatching rate, larval survival, adult emergence, no. of adults (males and females), susceptibility index, and growth index of Trogoderma granarium in six wheat genotypes during the free- and no-choice tests. Means in a column followed by different letters show significant differences (*p* < 0.01; Tukey’s HSD test) between the genotypes.

Test Type	Genotypes	Fecundity	Hatching (%)	Larval Survival (%)	Adult Emergence (%)	No. of Males	No. of Females	Susceptibility Index	Growth Index
Free-choice test	Arooj-2022	44.50 ± 0.76 ab	75.96 ± 1.62 ab	77.74 ± 1.65 a	80.03 ± 2.14 ab	10.17 ± 0.83 a	11.00 ± 0.58 a	6.55 ± 0.18 a	1.72 ± 0.05 a
	Dilkash-2021	42.83 ± 1.05 bc	77.97 ± 1.77 a	75.53 ± 1.49 a	82.16 ± 1.14 a	10.00 ± 0.37 a	10.67 ± 0.21 a	6.15 ± 0.13 ab	1.67 ± 0.04 a
	Akbar-2019	33.17 ± 0.83 e	68.30 ± 0.65 c	66.84 ± 0.90 c	70.28 ± 1.16 d	5.00 ± 0.26 c	5.67 ± 0.33 c	4.02 ± 0.10 d	1.20 ± 0.02 d
	Nawab-2021	46.67 ± 0.71 a	74.00 ± 1.33 abc	73.46 ± 2.22 ab	77.66 ± 1.53 abc	9.50 ± 0.34 a	10.17 ± 0.40 a	5.80 ± 0.04 b	1.51 ± 0.02 b
	Zincol-2016	36.00 ± 0.63 de	69.86 ± 1.41 bc	68.96 ± 1.19 bc	72.94 ± 1.55 cd	6.00 ± 0.37 bc	6.67 ± 0.21 bc	4.43 ± 0.07 d	1.28 ± 0.03 cd
	Bhakkar-2019	39.33 ± 0.88 cd	71.54 ± 1.49 bc	71.69 ± 0.68 abc	75.09 ± 0.76 bcd	7.33 ± 0.33 b	7.83 ± 0.31 b	4.98 ± 0.08 c	1.38 ± 0.02 bc
	F*_Statistic_*	39.8	6.79	7.89	9.50	23.3	38.6	85.7	41.3
	*p_value_*	≤0.01	≤0.01	≤0.01	≤0.01	≤0.01	≤0.01	≤0.01	≤0.01
	HSD at 0.01	3.54	6.11	6.23	6.21	1.97	1.56	0.47	0.14
	%CVs	4.99	4.77	4.90	4.63	14.01	10.25	4.98	5.55
No-choice test	Arooj-2022	40.83 ± 0.60 a	74.63 ± 1.39 abc	79.92 ± 1.59 a	81.40 ± 1.19 a	9.33 ± 0.42 a	10.50 ± 0.43 a	6.42 ± 0.13 a	1.75 ± 0.04 a
	Dilkash-2021	39.33 ± 0.71 ab	78.46 ± 1.13 a	77.26 ± 1.27 ab	79.04 ± 1.38 ab	9.00 ± 0.26 a	9.83 ± 0.31 a	5.96 ± 0.14 b	1.61 ± 0.05 ab
	Akbar-2019	32.50 ± 0.62 e	70.20 ± 1.64 cd	67.30 ± 1.73 d	70.71 ± 2.55 c	5.17 ± 0.31 c	5.67 ± 0.21 d	4.05 ± 0.10 e	1.20 ± 0.05 e
	Nawab-2021	37.67 ± 0.88 bc	76.92 ± 1.48 ab	74.92 ± 1.38 abc	76.94 ± 1.58 abc	8.17 ± 0.31 a	8.50 ± 0.22 b	5.48 ± 0.08 c	1.50 ± 0.03 bc
	Zincol-2016	34.17 ± 0.48 de	68.33 ± 1.41 d	69.97 ± 1.86 cd	72.38 ± 1.32 bc	5.67 ± 0.33 bc	6.17 ± 0.31 cd	4.32 ± 0.09 e	1.27 ± 0.02 de
	Bhakkar-2019	36.00 ± 0.58 cd	72.24 ± 1.33 bcd	72.37 ± 1.61 bcd	74.43 ± 0.90 bc	6.67 ± 0.33 b	7.33 ± 0.21 bc	4.84 ± 0.02 d	1.37 ± 0.02 cd
	F*_Statistic_*	22.9	7.77	8.71	6.66	28.2	44.9	88.6	32.3
	*p_value_*	≤0.01	≤0.01	≤0.01	≤0.01	≤0.01	≤0.01	≤0.01	≤0.01
	HSD at 0.01	2.83	6.05	6.82	6.78	1.42	1.26	0.43	0.16
	%CVs	4.38	4.69	5.27	5.09	11.04	8.94	4.71	6.25

**Table 3:** Damage parameters (mean ± SE) in different wheat genotypes caused by *R. dominica* after 30, 60, and 90 days of storage. Means in a column followed by different letters show significant differences (*p* < 0.01; Tukey’s HSD test) between the genotypes.

Genotypes	30DAS					60DAS					90DAS				
	NU	ND	DG (%)	FW (g)	WL (%)	NU	ND	DG (%)	FW (g)	WL (%)	NU	ND	DG (%)	FW (g)	WL (%)
Arooj-2022	536.50 ± 3.56 d	7.33 ± 0.67 a	1.35 ± 0.12 a	0.09 ± 0.01 a	0.66 ± 0.05 a	506.17 ± 1.51 d	37.67 ± 2.50 a	6.91 ± 0.42 a	0.18 ± 0.02 a	2.49 ± 0.14 a	397.83 ± 2.12 d	146.00 ± 1.83 a	26.84 ± 0.19 a	0.80 ± 0.02 a	7.95 ± 0.12 a
Dilkash-2021	556.50 ± 1.73 c	6.17 ± 0.48 a	1.10 ± 0.08 ab	0.08 ± 0.01 ab	0.51 ± 0.03 ab	529.17 ± 3.49 c	33.50 ± 1.65 ab	5.96 ± 0.31 ab	0.16 ± 0.02 ab	2.05 ± 0.13 a	427.50 ± 2.26 c	135.17 ± 2.52 b	24.02 ± 0.41 b	0.74 ± 0.02 ab	7.29 ± 0.13 b
Akbar-2019	600.50 ± 2.29 a	1.50 ± 0.43 d	0.25 ± 0.07 e	0.03 ± 0.01 c	0.10 ± 0.03 e	593.00 ± 2.07 a	9.00 ± 0.93 d	1.49 ± 0.15 d	0.07 ± 0.01 d	0.48 ± 0.08 d	522.33 ± 2.33 a	79.67 ± 1.98 e	13.23 ± 0.30 f	0.50 ± 0.03 d	3.62 ± 0.09 f
Nawab-2021	586.00 ± 2.14 b	5.33 ± 0.61 ab	0.90 ± 0.10 bc	0.06 ± 0.01 abc	0.41 ± 0.05 bc	562.67 ± 2.19 b	28.67 ± 1.33 bc	4.85 ± 0.22 bc	0.14 ± 0.01 abc	1.52 ± 0.09 b	463.17 ± 2.71 b	128.17 ± 2.32 b	21.68 ± 0.37 c	0.68 ± 0.02 bc	6.51 ± 0.22 c
Zincol-2016	611.33 ± 2.32 a	2.17 ± 0.31 cd	0.35 ± 0.05 de	0.04 ± 0.01 bc	0.15 ± 0.01 de	598.00 ± 2.53 a	15.50 ± 1.34 d	2.53 ± 0.21 d	0.09 ± 0.01 cd	0.95 ± 0.08 c	512.00 ± 2.53 a	101.50 ± 1.34 d	16.55 ± 0.21 e	0.58 ± 0.02 cd	4.32 ± 0.11 e
Bhakkar-2019	577.67 ± 2.78 b	4.00 ± 0.37 bc	0.69 ± 0.06 cd	0.05 ± 0.01 abc	0.30 ± 0.03 cd	558.83 ± 3.53 b	22.83 ± 1.56 c	3.93 ± 0.28 c	0.11 ± 0.01 bcd	1.22 ± 0.10 bc	463.67 ± 2.80 b	118.00 ± 2.11 c	20.29 ± 0.34 d	0.63 ± 0.02 c	5.68 ± 0.14 d
F Statistic	120	21.5	25.3	4.23	35.3	180	45.2	54.3	8.58	47.6	374	139	248	21.6	149
*p* value	≤0.01	≤0.01	≤0.01	≤0.01	≤0.01	≤0.01	≤0.01	≤0.01	≤0.01	≤0.01	≤0.01	≤0.01	≤0.01	≤0.01	≤0.01
HSD at 0.01	10.91	2.12	0.36	0.05	0.16	11.43	6.98	1.20	0.07	0.46	10.63	8.82	1.35	0.10	0.60
%CVs	1.07	27.37	26.88	47.05	24.92	1.17	16.21	15.93	30.45	17.91	1.30	4.25	3.77	8.80	5.76

DAS, days after storage; NU, number of undamaged grains; ND, number of damaged grains; DG, damaged grain; FW, frass weight; WL, weight loss.

**Table 4:** Damage parameters (mean ± SE) in different wheat genotypes caused by *T. granarium* after 30, 60, and 90 days of storage. Means in a column followed by different letters show significant differences (*p* < 0.01; Tukey’s HSD test) between the genotypes.

Genotypes	30DAS					60DAS					90DAS				
	NU	ND	DG (%)	FW (g)	WL (%)	NU	ND	DG (%)	FW (g)	WL (%)	NU	ND	DG (%)	FW (g)	WL (%)
Arooj-2022	502.33 ± 3.17 d	52.17 ± 0.79 a	9.41 ± 0.13 a	0.22 ± 0.02 a	2.09 ± 0.07 a	500.83 ± 3.12 d	53.67 ± 0.99 a	9.68 ± 0.16 a	0.24 ± 0.02 a	2.10 ± 0.09 a	456.33 ± 3.06 d	98.17 ± 1.40 a	17.70 ± 0.23 a	0.59 ± 0.01 a	3.93 ± 0.15 a
Dilkash-2021	518.17 ± 2.41 c	48.67 ± 0.67 b	8.59 ± 0.13 b	0.18 ± 0.02 ab	1.86 ± 0.05 ab	516.83 ± 2.40 c	50.00 ± 0.77 ab	8.82 ± 0.15 b	0.20 ± 0.02 ab	1.88 ± 0.10 ab	475.17 ± 2.60 c	91.67 ± 1.50 b	16.17 ± 0.27 b	0.56 ± 0.01 ab	3.54 ± 0.14 ab
Akbar-2019	569.67 ± 3.23 a	26.00 ± 0.63 f	4.37 ± 0.12 f	0.06 ± 0.01 d	1.17 ± 0.05 e	569.17 ± 3.09 a	26.50 ± 0.56 e	4.45 ± 0.11 f	0.08 ± 0.01 d	1.18 ± 0.04 d	534.17 ± 3.38 a	61.50 ± 0.99 f	10.33 ± 0.20 f	0.40 ± 0.01 e	2.12 ± 0.08 d
Nawab-2021	538.33 ± 2.53 b	44.83 ± 0.79 c	7.69 ± 0.15 c	0.14 ± 0.02 bc	1.68 ± 0.06 bc	536.83 ± 2.60 b	46.33 ± 0.99 b	7.95 ± 0.18 c	0.16 ± 0.02 bc	1.70 ± 0.10 bc	497.33 ± 2.47 b	85.83 ± 1.28 c	14.72 ± 0.22 c	0.51 ± 0.01 bc	3.27 ± 0.16 b
Zincol-2016	572.00 ± 2.92 a	33.50 ± 1.18 e	5.54 ± 0.20 e	0.08 ± 0.01 cd	1.34 ± 0.05 de	571.33 ± 2.84 a	34.17 ± 1.01 d	5.65 ± 0.17 e	0.11 ± 0.01 cd	1.35 ± 0.05 cd	535.67 ± 2.82 a	69.83 ± 1.22 e	11.53 ± 0.21 e	0.44 ± 0.01 de	2.53 ± 0.08 cd
Bhakkar-2019	535.67 ± 2.99 b	39.67 ± 0.67 d	6.90 ± 0.13 d	0.11 ± 0.01 cd	1.53 ± 0.04 cd	534.50 ± 3.15 b	40.83 ± 0.95 c	7.10 ± 0.18 d	0.14 ± 0.01 bcd	1.55 ± 0.09 bc	497.83 ± 3.40 b	77.50 ± 1.15 d	13.47 ± 0.24 d	0.48 ± 0.01 cd	3.02 ± 0.13 ab
F*_Statistic_*	91.4	147	168	15.0	40.8	94.6	131	150	15.3	17.5	112	118	148	29.4	27.9
*p_value_*	≤0.01	≤0.01	≤0.01	≤0.01	≤0.01	≤0.01	≤0.01	≤0.01	≤0.01	≤0.01	≤0.01	≤0.01	≤0.01	≤0.01	≤0.01
HSD at 0.01	12.43	3.48	0.63	0.07	0.23	12.39	3.85	0.69	0.07	0.35	12.81	5.45	0.98	0.06	0.54
%CVs	1.31	4.86	5.05	29.08	8.01	1.31	5.22	5.40	23.91	12.18	1.46	3.84	4.01	6.52	9.97

DAS, days after storage; NU, number of undamaged grains; ND, number of damaged grains; DG, damaged grain; FW, frass weight; WL, weight loss.

## Data Availability

All the data are present within the manuscript. Further inquiries can be directed to corresponding authors.
